# MFAP4 as a novel biomarker for predicting liver fibrosis and prognosis in infants with biliary atresia through the integration of bioinformatics with clinical data analysis

**DOI:** 10.3389/fped.2025.1611564

**Published:** 2025-05-26

**Authors:** Chunxiao Yang, Jianghua Zhan

**Affiliations:** ^1^ Graduate College, Tianjin Medical University, Tianjin, China; ^2^ Department of General Surgery, Zibo Municipal Hospital, Shandong, China; ^3^ Department of General Surgery, Tianjin Children’s Hospital, Tianjin, China

**Keywords:** biliary atresia, liver fibrosis, MFAP4, immunohistochemistry, PCR

## Abstract

**Background:**

Biliary atresia (BA) is a progressive obliteration of intrahepatic and extrahepatic bile ducts. Our study aimed to identify and validate hub genes that are differentially expressed in BA, and to investigate their relationship with liver fibrosis.

**Methods:**

The BA microarray datasets GSE46960 and GSE15235 were downloaded from the Gene Expression Omnibus (GEO) database. Clinical BA liver tissue samples were collected for quantitative reverse transcription-polymerase chain reaction (qRT-PCR) and western blotting (WB). Serum samples were analyzed for MFAP4 content using enzyme-linked immunosorbent assay (ELISA), and the corresponding clinical data of pediatric patients were collected. The relationship between serum MFAP4 levels and liver fibrosis, as well as prognosis, was investigated using ROC curve analysis. Furthermore, Kaplan–Meier (KM) curves were used to validate the prognostic significance of MFAP4 levels.

**Results:**

Through bioinformatics analysis and experimental validation, MFAP4 was identified as a hub gene. MFAP4 via PCR and immunohistochemistry is significantly upregulated in biliary atresia compared to total functional cyst liver tissue. Serum MFAP4 levels accurately reflects the degree of liver fibrosis in patients with BA. High serum MFAP4 levels predicted a poorer native liver survival rate one year after Kasai surgery.

**Conclusions:**

We discovered that MFAP4 is a crucial BA-associated gene, and validated its expression in BA. Furthermore, serum MFAP4 levels may serve as predictive markers for the degree of liver fibrosis and autologous liver survival after Kasai surgery.

## Introduction

Biliary atresia (BA) is a progressive obliteration of intrahepatic and extrahepatic bile ducts (1). If left untreated, persistent cholestasis results in progressive liver cirrhosis, ultimately leading to death around the age of 1 year (2). The Kasai procedure is the first-line treatment for BA (3), which can alleviate biliary obstruction and restore bile drainage in some children; however, it cannot prevent progressive fibrosis (4, 5).

Liver fibrosis represents a bottleneck in BA treatment. Despite extensive research into the mechanisms of liver fibrosis in BA from various perspectives such as immune injury and cholestasis (6–8), the etiology of BA-related liver fibrosis remains complex, with rapid progression and poor prognosis compared to other forms of chronic liver fibrosis. Unfortunately, no effective treatment has been identified to date (9, 10), and there is still no clear explanation for this phenomenon.

Therefore, we aimed to explore the relationship between BA and liver fibrosis from a genetic perspective. In this study, we collected BA microarray datasets from GEO and analyzed differential gene expression in conjunction with enrichment analyses to investigate the association between BA and liver fibrosis. We identified hub genes and validated their expression in relevant patient samples.

To identify differentially expressed genes associated with BA and liver fibrosis, we employed bioinformatics approaches to analyze GEO datasets pertinent to BA, culminating in the selection of a pivotal gene, MFAP4. The expression of this gene in BA samples was subsequently confirmed by immunohistochemical staining and PCR experiments. Furthermore, ELISA was used to quantify circulating MFAP4 levels, thereby investigating the correlation between this gene and clinical data in patients with BA. The outcomes of this study offer new perspectives on the diagnosis and treatment of BA.

## Methods

### Gene expression profile data

Gene expression data for BA were obtained from the GEO database, specifically the GSE46960 and GSE15235 datasets (11, 12), which were used in our study. Platform annotation files were employed to convert the “probe ids” in the expression matrices into “gene symbols”. The average value was calculated as the representative expression level for multiple probes corresponding to the same gene symbol. Data normalization and identification of differentially expressed genes (DEGs) associated with BA were performed using the limma package in the R software.

### Functional enrichment analysis of differentially expressed genes

Using the Gene Ontology (GO) database and Kyoto Encyclopedia of Genes and Genomes (KEGG) pathway database, we conducted enrichment analyses on the DEGs.

Using the “clusterProfiler” package in R software (13), we annotated the DEGs identified from the GSE46960 dataset related to biliary atresia. This process aimed to uncover all functional categories associated with these genes. We employed Fisher's Exact Test and multiple comparison test to calculate the significance level (*P*-value) and false discovery rate (FDR) for each functional category. Genes were considered significantly enriched in a particular function if they met the criterion of *p* < 0.05.

Using the KEGG database, we conducted an enrichment analysis of the DEGs within the signaling pathways. This was achieved using the Pathway Enrichment Analysis method, which annotated the DEGs obtained from the KEGG database with their respective pathways (pathway Annotation). This process identified all the signaling pathways (Pathways) in which the genes participate.

Subsequently, we used Fisher's Exact Test and multiple comparison tests to calculate the significance level (*P*-value) and false discovery rate (FDR) for each signaling pathway. These calculations enabled us to filter out the significant signaling pathways reflected by the genes, with the significance threshold set at *P*-value <0.05.

When performing enrichment analysis on expressed genes utilizing the Gene Set Enrichment Analysis (GSEA) database, the results of this analysis were subjected to validation procedures to identify and select the significant signaling pathways reflected by the genes. The criterion for determining significance is set at a *P*-value threshold of less than 0.05.

### Screening of core genes

By inputting the differentially expressed genes into the Search Tool for the Retrieval of Interacting Genes (STRING) database, a protein-protein interaction (PPI) network for these genes was constructed, with a confidence threshold set at >0.4. The PPI network was then visualized using Cytoscape (version 3.9.1), where the connectivity of protein nodes was calculated using the cytoHubba and MCODE plugins. Based on this analysis, the top 10 genes were selected as core genes. Additionally, proteomic modules exhibiting significant differences were filtered out, and core genes specific to BA were identified within these modules.

### Screening of genes highly correlated with core genes and functional enrichment analysis

Using the clusterProfiler package in R software (13), we screened genes with a high correlation to the selected core genes from datasets GSE46960 and GSE15235. The screening criteria were set to select genes with a correlation coefficient of |Cor|>0.7 and a *P*-value < 0.05. Subsequently, we performed enrichment analysis by combining GO, KEGG, and LogFC values. The Pathway Enrichment Analysis method was employed for pathway annotation, yielding all signaling pathways (pathways) in which the genes participate. Fisher's exact test and multiple comparison tests were used to calculate the significance level (*p*-value) and false discovery rate (FDR) for each signaling pathway. This allowed us to filter out the significant signaling pathways reflected by the genes, with a significance threshold of *P*-value <0.05.

### Patient characteristics

A retrospective analysis was conducted of serum samples and clinical data from 29 infants diagnosed with BA admitted to Tianjin Children's Hospital between January 2016 and December 2019. Liver tissue samples from 13 children with choledochal cysts admitted during the same period were randomly selected as controls. The baseline characteristics of all the patients are presented in Table 1. We selected 16 samples from 29 patients with biliary atresia and 8 samples from 13 patients with choledochal cyst for Quantitative Reverse Transcription Polymerase Chain Reaction (qRT-PCR) analysis. Liver fibrosis was assessed based on the modified Ishak staging system, which classifies liver fibrosis on a four-point scale with stage 4 defined as cirrhosis. We classified Ishak stages 1 and 2 of liver fibrosis as mild fibrosis, whereas Ishak stages 3 and 4 were designated as severe fibrosis. Based on the assessment of liver fibrosis, the BA cohort was stratified into two groups: 13 infants with mild fibrosis and 16 infants with severe fibrosis. There was no statistically significant difference in surgical age between the two groups (Table 2). Baseline biochemical analysis revealed no significant difference in the elevated levels of total bilirubin (TBIL), direct bilirubin (DBIL), gamma-glutamyl transpeptidase (GGT), and transaminases between the mild and severe fibrosis groups (Table 2).

**Table 1:** Demographic information and baseline laboratory data for BA group and CBD group.

Characteristics	BA	CBD	*P*-value
*n*	29	13	
The age of surgery, days, mean ± sd	61.828 ± 21.366	110 ± 19.209	<0.001
Sex, *n* (%)			0.555
Male	14 (33.3%)	5 (11.9%)	
Female	15 (35.7%)	8 (19%)	
TBIL, mean ± sd	184.37 ± 49.289	126.54 ± 16.024	<0.001
DBIL, mean ± sd	140.58 ± 40.696	82.923 ± 15.65	<0.001
ALT, median (IQR)	107 (84, 172)	53 (42, 62)	<0.001
AST, median (IQR)	188 (137, 320)	69 (61, 84)	<0.001
GGT, median (IQR)	370 (167, 636)	184 (171, 198)	0.028

**Table 2:** Demographic information and baseline laboratory data for mild fibrosis group BA and severe fibrosis group BA cohorts.

Characteristics	Mild fibrosis group	Severe fibrosis group	*P*-value
*n*	13	16	
The age of surgery, days, mean ± sd	57.154 ± 21.969	65.625 ± 20.778	0.297
Sex, *n* (%)			0.272
Female	5 (17.2%)	10 (34.5%)	
Male	8 (27.6%)	6 (20.7%)	
TBIL,umol/L, mean ± sd	186.1 ± 43.318	182.97 ± 55.036	0.868
DBIL,umol/L, mean ± sd	138.09 ± 43.637	142.59 ± 39.478	0.773
ALT, U/L, median (IQR)	107 (52, 172)	107.5 (97.25, 172.75)	0.469
AST, U/L, median (IQR)	252 (123, 345)	181.5 (141.5, 310.25)	0.982
GGT, U/L, median (IQR)	207 (147, 378)	432 (232.75, 751.25)	0.263

This study was approved by the ethics committees of Tianjin Children's Hospital (2022-SYYJCYJ-008) and informed consent was obtained from each participant's guardian. We confirm that all research processes comply with the Helsinki Declaration revised in 2013.

### Immunohistochemistry and the interpretation of liver fibrosis

After tissue embedding, sectioning, and HE staining, histological microarrays were constructed. Histological microarrays were placed in an oven at 70°C for 1 h, followed by alcohol dehydration after routine xylene dewaxing. Subsequently, they were repaired in EDTA buffer with a pH of 8.0 at a high temperature of 100°C for 5 min, repeating this process four times, and allowed to cool naturally. Endogenous peroxidase activity was inhibited using 0.3% hydrogen peroxide and methanol (30 min). The samples were rinsed three times with PBS for 10 min each. Goat serum was used for blocking at room temperature for 1 h, after which the serum was discarded. Next, the MFAP4 rabbit polyclonal antibody (Proteintech) was diluted at a ratio of 1:400, dripped onto the slides, and incubated overnight at 4°C (not exceeding 16 h). The secondary antibody was applied in a humidified chamber at room temperature for 40 min the next day, and a color development reaction was performed using diaminobenzidine (DAB) staining. Subsequently, the slides were stained, fixed with hematoxylin and counterstained with aqueous ammonia. The staining results for all slides were evaluated by two professional pathologists.

The sections were observed under a Nikon optical microscope (Germany) at a total magnification of 100×. For each section, five random areas were selected, and the integrated optical density (IOD) and area values of each random area were measured. The mean density was calculated as mean density = IOD/area, which reflects the concentration of the target protein per unit area. Finally, the average of the mean density values from five random areas of each section was taken as the value for that section.

The interpretation of liver fibrosis was conducted independently by two pathologists, who evaluated the presence and severity of fibrosis based on both gross and microscopic examinations. The final diagnosis, relied solely on microscopic observations and involved the assessment of HE-stained pathological sections of BA liver tissue specimens. These were evaluated by specialized pathologists according to Ohkuma's grading system, which categorizes fibrosis into five distinct levels: grade 0, absence of fibrosis; Grade I, mild fibrosis in the portal areas; Grade II, mild bridging fibrosis adjacent to the portal areas; Grade III, extensive bridging fibrosis; and Grade IV, cirrhosis with pseudolobule formation. For the purpose of this study, Grades I and II were collectively defined as the mild fibrosis group, whereas Grades III and IV were classified as the severe fibrosis group.

### Quantitative reverse transcription polymerase chain reaction (qRT-PCR)

Total RNA was extracted from the liver tissues of patients with BA and choledochal cyst using an RNA-easy Isolation Reagent and quantified using a spectrophotometer (USA). Complementary cDNA was synthesized from total RNA using the Master Mix Reverse Transcription. Amplification was performed on a Roche LightCycler 96 real-time fluorescent quantitative PCR system (Roche, Germany) using SYBR Green (Sangon Biotech, China). Relative gene expression levels were normalized to those of β-actin and calculated using the 2−ΔΔCt method. Three technical replicates were performed for each sample. The primer sequences used were: GAPDH-F: 5′-CTCCATCCTGGCCTCGCTGT-3′, GAPDH-R: 5′-GCTGTCACCTTCACCGTTCC-3′, MFAP4-F: 5′-GGCTCAGTAAGTTTCTTCCGCG-3′, and MFAP4-R: 5′-CCAAGTCCACTCGCAGCTCATA-3′.

### Serum MFAP4 assay

Liver tissue samples were randomly selected from 29 children with biliary atresia admitted to Tianjin Children's Hospital between January 2016 and December 2019.

The study was conducted using serum samples and clinical data from 29 infants with biliary atresia admitted to Tianjin Children's Hospital between January 2016 and December 2019. Based on the assessment of liver fibrosis, the cohort was stratified into two groups: 13 infants with mild fibrosis and 16 infants with severe fibrosis. Serum samples were stored at −80°C. The MFAP4 concentration was measured in duplicates using an ELISA kit (catalog number m1950502V; Mlbio) following the manufacturer's protocol. Samples that exceeded the calibration range were remeasured after 1:10 dilution.

## Results

### Screening and functional analysis of differentially expressed genes in biliary atresia

**Figure 1:**
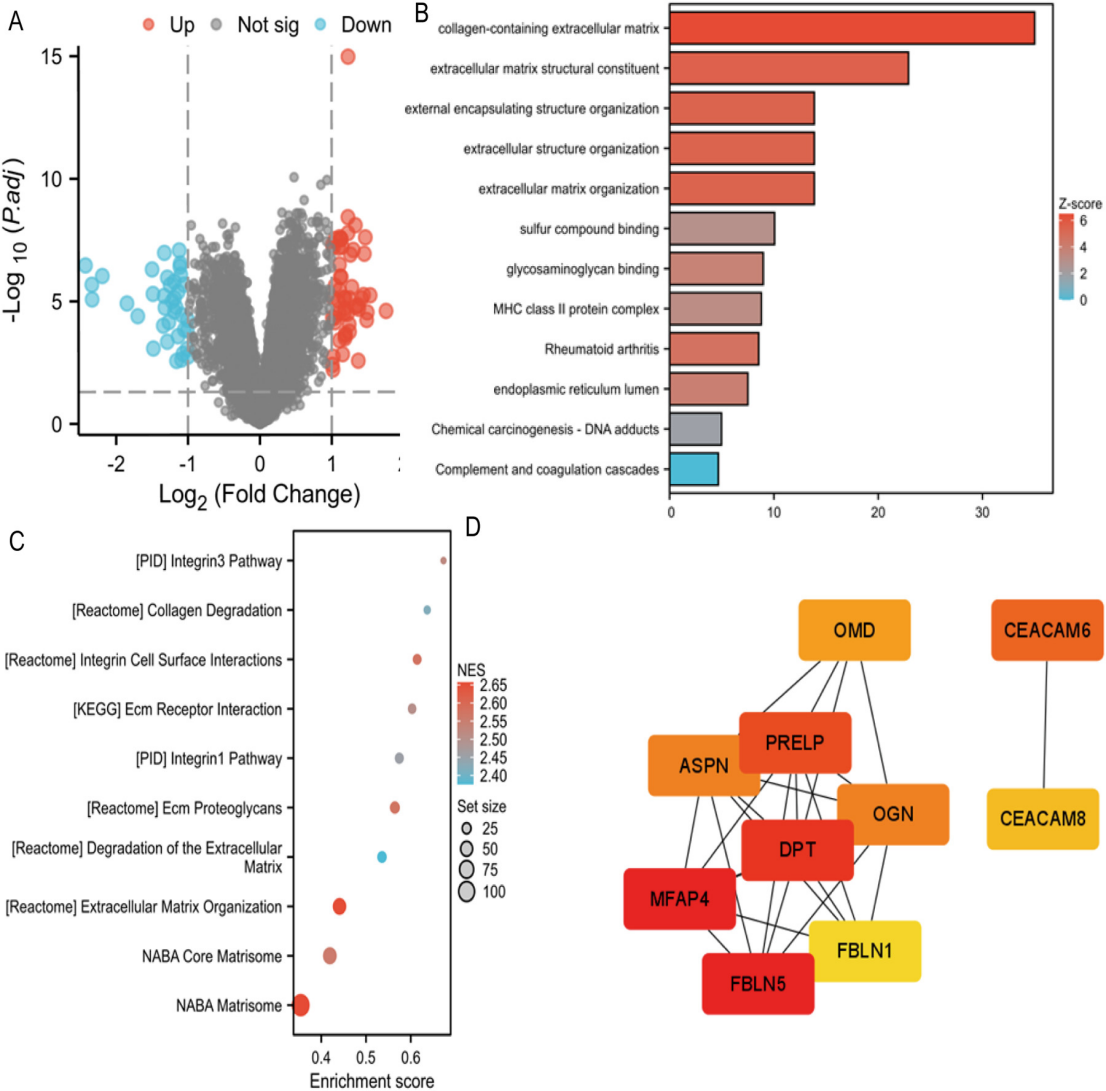
**(A)** Volcano map of differentially expressed genes in GSE46960. **(B)** GOKEGG combined with FC analyses in GSE46960. Z-score, normalized enrichment score. **(C)** GSEA analyses in GSE46960. NES, normalized enrichment score. **(D)** GSEA analyses in GSE15235. NES, normalized enrichment score.

The dataset GSE46960 examined liver tissues from 64 children with BA and 7 normal infants. Differential gene screening was performed based on the RNA sequencing results, with the screening criteria set as fold change (FC) value |FC|>1 and *P*-value < 0.05. A total of 93 differentially expressed genes were identified, including 55 upregulated and 38 downregulated genes (Figure 1A). GOKEGG combined with logFC gene functional enrichment analysis showed that the differentially expressed genes were mainly enriched in collagen-containing extracellular matrix, extracellular matrix structural constituent, external encapsulating structure organization, extracellular structure organization, extracellular matrix organization, sulfur compound binding, glycosaminoglycan binding, MHC class II protein complex, rheumatoid arthritis, endoplasmic reticulum lumen, chemical carcinogenesis - DNA adduct, and complement and coagulation cascades (Figure 1B). GSEA enrichment analysis revealed that the expressed genes were mainly enriched in the Integrin3 pathway, collagen degradation, integrin cell surface interaction, ECM-receptor interaction, Integrin1 pathway, ECM proteoglycan, degradation of extracellular matrix, extracellular matrix organization, NABA core matrisome, and NABA matrisome (Figure 1C).

### Construct a PPI (protein-protein interaction) network for biliary atresia to screen for core genes

**Figure 2:**
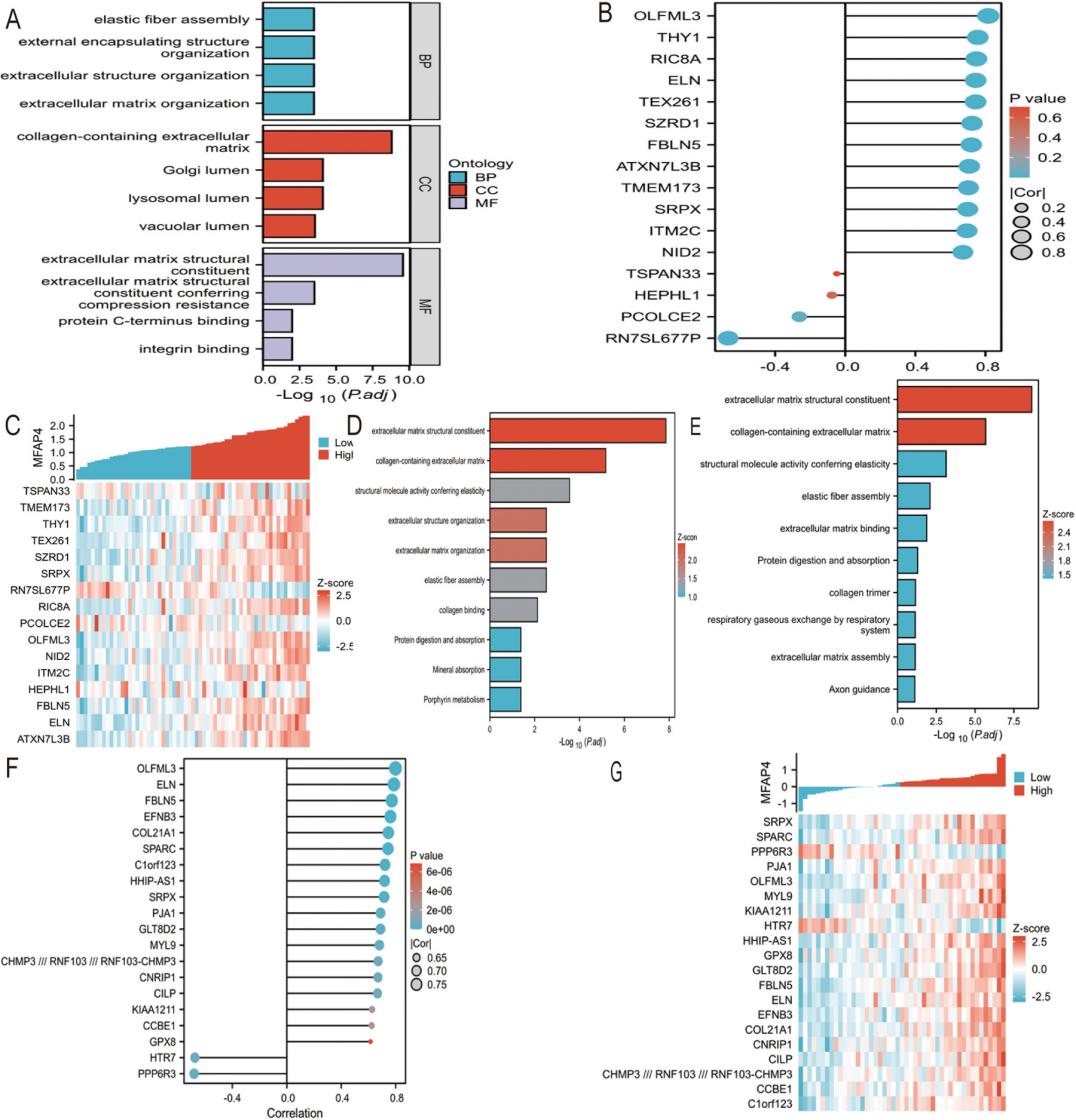
**(A)** PPI network of BA were visualized. Red genes indicate higher PPI network score. **(B)** GO functional enrichment analyses of hub genes. **(C)** Screening genes closely related to MFAP4 in GSE46960. Cor indicates correlation coefficient. **(D)** Screening genes closely related to MFAP4 in GSE46960. Z-score indicates correlation score. **(E)** GOKEGG combined with FC analyses of genes closely related to MFAP4 in GSE46960. Z-score, normalized enrichment score. (F) Screening genes closely related to MFAP4 in GSE15235. Cor indicates correlation coefficient. **(G)** Screening genes closely related to MFAP4 in GSE15235. Z-score indicates correlation score. **(H)** GOKEGG combined with FC analyses of genes closely related to MFAP4 in GSE15235. Z-score, normalized enrichment score.

Using Stringdb, a protein interaction (PPI) network of differentially expressed genes regulating biliary atresia was constructed. Based on the node connectivity within the PPI network (Figure 1D), 10 core genes regulating biliary atresia were identified: MFAP4, FBLN5, DPT, PRELP, CEACAM6, OMD, APSN, OGN, CEACAM8, and FBLN1. Among these, MFAP4 has emerged as the top-ranked core gene. A protein interaction module containing the core gene MFAP4 was obtained using the MCODE plugin. Subsequent GO and KEGG analyses revealed that the genes within this module were primarily enriched in extracellular matrix, elastic fibers, integrin binding, and other related functions (Figure 2A).

### Functional analysis of genes with high correlation to core genes

Based on the RNA sequencing results from dataset GSE46960, a batch correlation analysis was conducted to identify genes highly correlated with the core gene MFAP4. The screening criteria were set to include genes with a correlation coefficient of |Cor|>0.7 and a *P*-value <0.05. A total of 16 genes were identified as having high correlation with MFAP4, namely: TSPAN3, TMEM173, THY1, TEX261, SZRD1, SRPX, RN7SL677P, RIC8A, PCOLCE, OLFML3, NID2, ITM2C, HEPH, FBLN5, ELN, and ATXN7L3B (Figures 2B,C). A combination of GO and KEGG analyses with logFC gene function enrichment was performed on these genes, revealing enrichment in extracellular matrix structural constituents, collagen-containing extracellular matrix, structural molecule activity conferring elasticity, extracellular matrix organization, elastic fiber component, collagen binding, protein digestion and absorption, mineral absorption, and porphyrin metabolism (Figure 2D).

Based on the RNA sequencing results from dataset GSE15235, a batch correlation analysis was performed to identify genes with a high correlation to the core gene MFAP4. The screening criteria were set to include genes with a correlation coefficient of |Cor|>0.7 and a *P*-value <0.05. Twenty genes were identified as having a high correlation with MFAP4, namely: SRPX, SPARC, PPP6R3, PJA1, OLFML3, MYL9, KIAA1211, HTR7, HHIP-AS1, GPX8, GLT8D2, FBLN5, ELN, EFNB3, COL21A1, CNRIP1, CILP, CCBE1, CHMP3// RNF103// RNF103-CHMP3, and C1orf123 (Figures 2E,F). A combination of GO and KEGG analyses with logFC gene function enrichment was conducted on these genes, revealing enrichment in extracellular matrix structural constituents, collagen-containing extracellular matrix, structural molecule activity conferring elasticity, elastic fiber component, extracellular matrix binding, protein digestion and absorption, collagen trimer, respiratory gas exchange by the respiratory system, extracellular matrix organization, and axon guidance (Figure 2G).

### Outcomes of MFAP4 measured in human liver tissues with biliary atresia and choledochal cyst

In this study, immunohistochemistry was used to examine the differential expression of MFAP4 in human liver tissues affected by BA and choledochal cyst (CBD).

**Figure 3:**
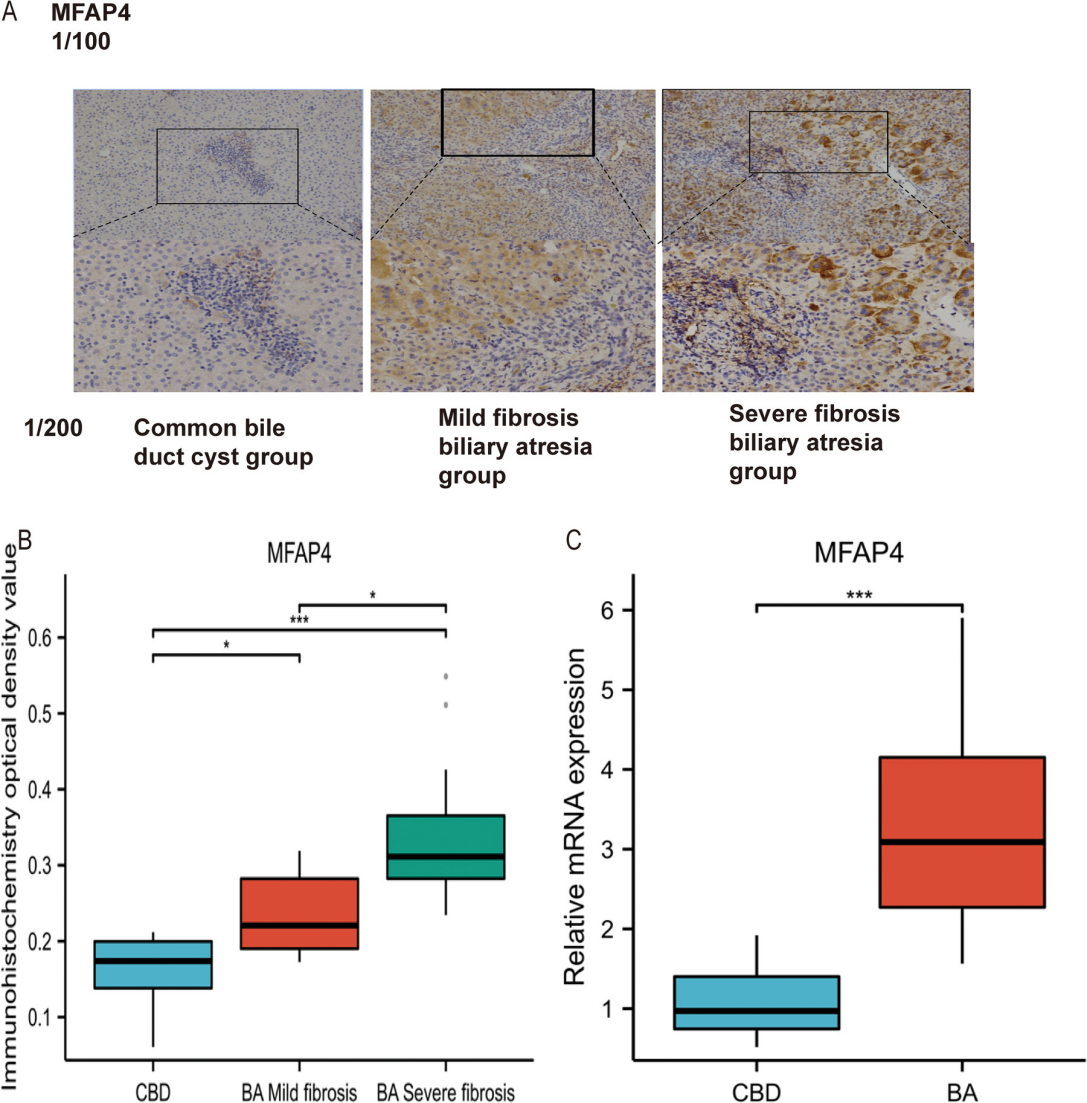
**(A)** Identification and validation of MFAP4 in Immunohistochemistry. **(B)** Validation of differential expression of MFAP4 in BA and CBD by Immunohistochemistry. **(C)** Validation of differential expression of MFAP4 in BA and CBD by qRT-PCR.

We assessed the localization and expression of MFAP4 using immunohistochemical staining (Figure 3A). The results demonstrated that MFAP4 is localized in the extracellular matrix but is also expressed in the cytoplasm. Notably, the expression of MFAP4 was higher in liver fibrosis tissues from patients compared than in those with choledochal cyst. Both parenchymal and non-parenchymal cells in the portal area expressed MFAP4, with a significant increase in MFAP4 expression observed in non-parenchymal cells (predominantly hepatic stellate cells) surrounding the portal area in the BA group compared to the choledochal cyst group.

Statistical analysis revealed significant differences in MFAP4 expression among CBD group, BA with mild fibrosis group, and BA with severe fibrosis group (*p* < 0.05, Figure 3B). Furthermore, MFAP4 expression was positively correlated with the pathological stage of liver fibrosis. Based on these findings, we speculated that MFAP4 plays a role in liver fibrosis.

RT-qPCR results suggested that both COL1A1 and MFAP4 were significantly upregulated in biliary atresia compared to the total functional cyst liver tissue (*P* < 0.05, Figure 3C).

### MFAP4 serum levels in the cohorts and predicted liver fibrosis and Kasai portoenterostomy (KPE) outcomes

**Figure 4:**
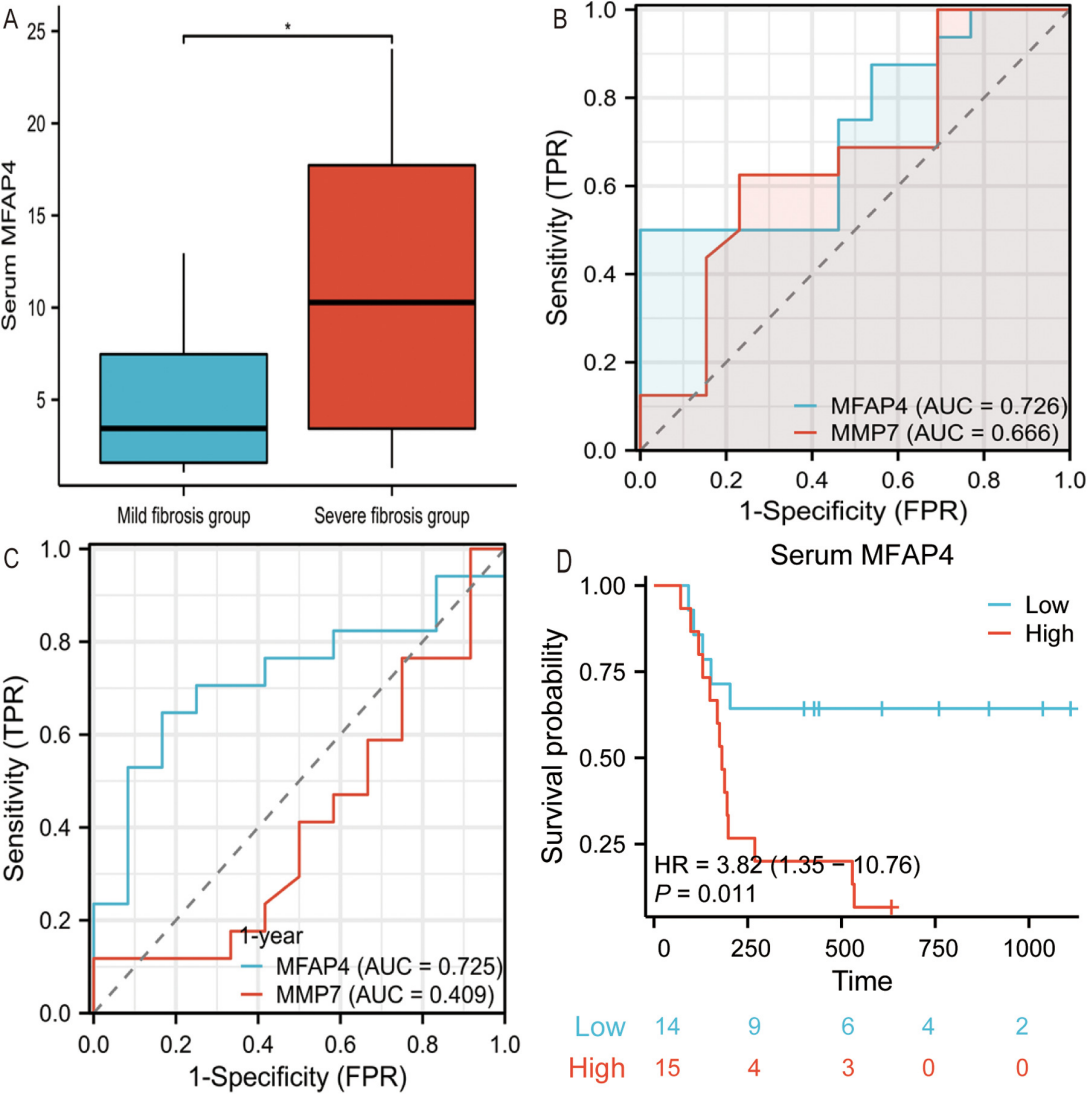
**(A)** Serum MFAP4 concentrations in mild fibrosis BA patients and severe fibrosis BA patients. **(B)** Comparison of MFAP4 performance with MMP7 differentiating mild liver fibrosis from severe liver fibrosis in BA patients. **(C)** Shows the AUC of MFAP4 and MMP-7 in the predicting survival of native liver one year after Kasai procedure. **(D)** Kaplan-Meier native liver survival curves according to serum MFAP4 for all BA patients.

Patients with BA were categorized into mild and severe fibrosis groups based on the degree of liver fibrosis. Compared with the BA mild fibrosis group, the median serum MFAP4 concentration in the BA severe fibrosis group was twice as high, demonstrating a statistically significant difference between the two groups (*P* < 0.05, Figure 4A).

When using serum MFAP4 and MMP7 to predict the severity of liver fibrosis in BA patients, higher serum MFAP4 levels were found to be superior in predicting severe liver fibrosis [AUC = 0.726] (*P* < 0.05, Figure 4B), compared to MMP7 [AUC = 0.666], indicating that serum MFAP4 more accurately reflects the degree of liver fibrosis in BA patients. In time-dependent ROC analysis, high serum MFAP4 predicted a poorer native liver survival rate one year after Kasai surgery [AUC = 0.725] (*P* < 0.05, Figure 4C), outperforming MMP7 [AUC = 0.409].We employed Kaplan–Meier survival curves to compare native liver survival rates and calculate hazard ratios (HRs). High serum MFAP4 levels were significantly associated with reduced native liver survival rates, and emerged as a highly significant risk factor for liver transplantation or death during follow-up HR: 3.82 (95% CI, 1.35–10.06), *p* = 0.011 (*P* < 0.05, Figure 4D).

## Discussion

BA is a severe biliary disease unique to infants and is characterized by progressive fibrotic occlusion that ultimately leads to liver cirrhosis and failure (14, 15). Based on GEO datasets, this study screened for the hub gene MFAP4 and verified its expression in BA using qPCR and immunohistochemical experiments.

MFAP4, also known as 36-kDa microfibril-associated glycoprotein (MAGP-36) (16), contains an RGD sequence in its protein structure that recognizes specific subsets of integrin receptors. MFAP4 is significantly upregulated in liver tissues with cirrhosis (17, 18), and its expression colocalizes with *α*-smooth muscle actin (a myofibroblast marker) in fibrotic livers (19). However, no previous studies have investigated MFAP4 expression in patients with BA.

### Bioinformatics analysis

We analyzed different datasets (GSE46960 and GSE15235) using various enrichment analysis methods. Through the study of the dataset GSE46960, we found that the differentially expressed genes in BA were mainly enriched in extracellular matrix-related pathways, monocyte chemotaxis, and positive regulation of the ERK1 and ERK2 cascade. Organ fibrosis is defined as the excessive deposition of connective tissue (20). Therefore, the enrichment of extracellular matrix pathways is closely related to fibrosis. The enrichment of differentially expressed BA genes in extracellular matrix-related pathways suggests that BA may promote liver fibrosis at the gene expression level.

This was further confirmed by GSEA functional enrichment analysis of the GSE15235 dataset. The major enriched pathways identified by GSEA included ECM-related, collagen-related, and integrin-related pathways. Integrins transduce mechanical and biochemical signals, promoting ECM assembly and remodeling (21). The enriched pathways of differentially expressed genes in BA suggest that the expression of differential genes may contribute to the development of liver fibrosis.

### Expression of MFAP4 in human liver tissues with biliary atresia and choledochal cyst

Through bioinformatics analysis of public BA datasets, we considered MFAP4 as a relevant gene in BA. To further validate MFAP4 expression in BA patient specimens, qRT-PCR and immunohistochemistry were performed, and the results showed that MFAP4 expression in BA was consistent with our hypothesis. Thus, MFAP4 has emerged as a potentially crucial gene in the mechanisms underlying BA.

### Analysis of the relationship between serum MFAP4 levels, liver fibrosis and KPE outcomes

MFAP4 serves as a surrogate for gene expression levels in serum, facilitating its convenient measurement, which potentially enables a more accessible assessment of liver fibrosis severity using serum biomarkers in clinical practice. Currently, MFAP4 accurately predicts the degree of liver fibrosis secondary to hepatitis and alcoholic liver disease (22). Our study reports significant differences in serum MFAP4 levels among pediatric patients with biliary atresia (BA) stratified by liver fibrosis stages, underscoring the ease of MFAP4 detection in serum and its potential to streamline fibrosis assessment.

MMP7, a frequently employed biomarker in BA, despite significant variations in threshold levels among studies due to different measurement methods (23), is intimately correlated with the degree of liver fibrosis (24, 25). Our study demonstrated that MFAP4 outperformed MMP7 in accurately assessing the severity of liver fibrosis in patients with BA.

Notably, we found that serum MFAP4 levels can predict the native liver survival rate one year after Kasai surgery. A significant factor contributing to poor prognosis post-Kasai is the failure of liver fibrosis to resolve or even its progression. Elevated serum MFAP4 levels indicate a more severe degree of liver fibrosis, which may lead to decreased autologous liver survival. This notion was further validated using Kaplan–Meier survival curve analysis.

A limitation of this study is that no *in vivo* experiments were conducted to further validate the regulatory role of MFAP4 in liver fibrosis in BA. Therefore, we need to conduct additional cellular functional experiments are required for further verification.

## Conclusion

In summary, we identified MFAP4 as an important gene associated with liver fibrosis and the prognosis of BA. To the best of our knowledge, this is the first report of a relationship between MFAP4 and BA has been discovered at the genetic level. Furthermore, serum MFAP4 levels may serve as a predictive marker for the degree of liver fibrosis and autologous liver survival after Kasai surgery. Our findings provide a theoretical basis for the early assessment of BA fibrosis and prognosis in the future.

## Data Availability

The datasets GSE46960 and GSE15235 used in the current study are available in the GEO repository (https://www.ncbi.nlm.nih.gov/geo/) or from the corresponding author upon reasonable request.
